# Wild bats hunt insects faster under lit conditions by integrating acoustic and visual information

**DOI:** 10.1073/pnas.2515087122

**Published:** 2025-09-08

**Authors:** Laura Stidsholt, Mara Zebele, Carolin Scholz, Christian C. Voigt

**Affiliations:** ^a^Department of Evolutionary Ecology, Leibniz Institute for Zoo and Wildlife Research, Berlin 10315, Germany; ^b^Section for Zoophysiology, Department of Biology, Aarhus University, Aarhus 8000, Denmark; ^c^Research Group Evolutionary Ecology, Institute of Biochemistry and Biology, University of Potsdam, Potsdam 14476, Germany

**Keywords:** predator prey interaction, multimodal sensing, biologging

## Abstract

Animals can improve their decision-making abilities by integrating information from multiple senses, which is especially beneficial when living in fluctuating environments. However, understanding how wild predators may use multimodal sensing when hunting prey in split-second interactions remains largely unexplored. As nocturnal hunters, bats rely on echolocation to navigate and to locate evasive prey, yet they have retained functional vision, despite the associated costs. We therefore hypothesized that bats use vision to enhance sensory redundancy when commuting and tracking small insects. To test this, we equipped 21 wild common noctule bats (*Nyctalus noctula*) with high-resolution light, sound, and motion sensor loggers and measured their echolocation and movements while commuting and foraging in both dark and lit environments. When commuting, the bats maintained consistent echolocation sampling across light levels. However, when tracking prey in illuminated environments, the bats emitted calls with half the rate and with 7 dB higher call levels compared to in dark conditions, but at much faster approach speeds (from 5.2 in darkness to 7.9 m/s in lit conditions). This suggests that, in illuminated environments, hunting bats integrate acoustic and visual information, resulting in more efficient approaches to prey. Our findings demonstrate how a wild sensory specialist predator uses multimodal sensing to hunt efficiently in highly dynamic resource landscapes.

Bats can use vision (1) for short- and long-range orientation (2, 3), yet their night-adapted eyes have a low visual acuity, resulting in reduced range and resolution as light levels decrease. This has led to the hypothesis that bats do not use vision for hunting (4). However, many bats hunt insects in the early hours of the night in low light conditions, for which their vision is optimized (3), and rely on very weak prey echoes sampled at discrete time intervals by their biosonar. This makes bimodal sensing potentially highly beneficial for improving foraging success. To investigate whether and how these predators integrate vision and echolocation, we compared the movement and sensory patterns across behaviors (i.e., commuting vs. prey tracking) and light levels (i.e., dark vs. light).

## Results and Discussion

### Common Noctules Do Not Change Echolocation Behavior When Commuting in Lit Environments.

**Fig. 1.**
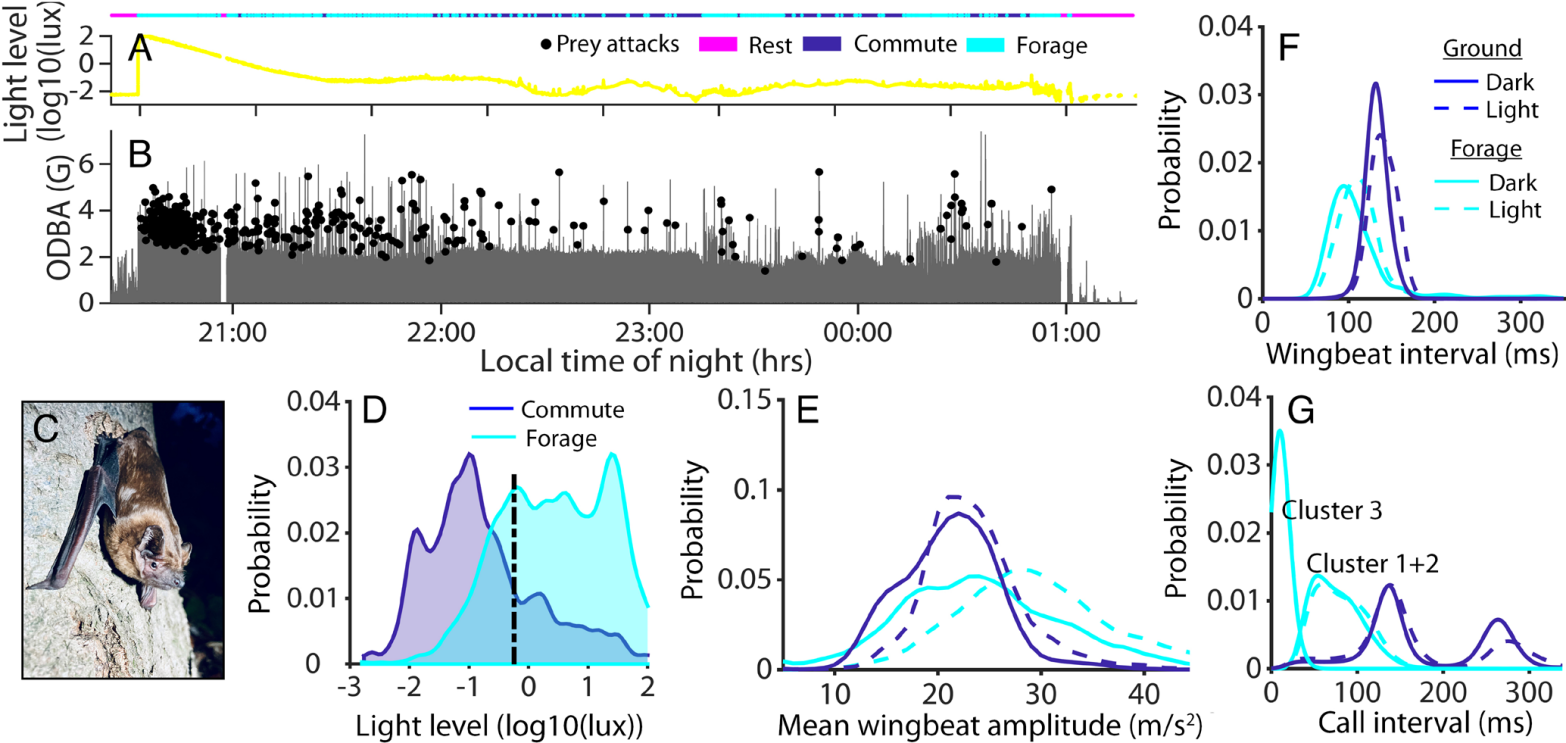
Flight activity as a function of experienced light levels of common noctules. Light levels (*A*) and nightly activity levels [i.e., rest (magenta), commuting (blue), and foraging (cyan)] were measured as the overall dynamic body acceleration (ODBA) with foraging attempts (black circles) plotted on top (*B*). The common noctules (*C*) experienced different light levels according to behavior (mean = dashed black line). n = 21 bats. Mean wingbeat amplitude (*E*), frequency (*F*), and call intervals (*G*) during commuting (blue) and foraging (cyan) and in light (dashed lines) compared to in dark (solid lines).

We first predicted that when visual information was available in lit conditions, bats would sample acoustic information at a reduced rate during commuting flights. However, we found that bats in lit conditions called more slowly (i.e., the mean call interval increased for both peaks of the bimodally distributed calls, Fig. 1*I*). We also found that they used slower wingbeat frequencies (i.e., wingbeat intervals increased from 131 ± 10 ms in dark to 139 ± 13 ms in lit conditions, LMM, *P* < 0.0001, model 1, Fig. 1*G*) at similar wingbeat stroke amplitudes (22.3 (SE: 1.15) vs. 24.4 (SE: 0.12) m/s^2^ in lit conditions, model 2, LMM, *P* < 0.001) (Fig. 1*H*, dark blue). The relatively small decrease in echolocation sampling rate during commuting in lit conditions is likely a passive consequence of slower wingbeat frequencies, rather than driven by the need for less echoic sensory input. The slower wingbeats in lit conditions may reflect faster flight speeds in light (5, 6), although the relationship between wingbeat frequency and amplitude can be complex and dependent on flight mode and speed. (7) Nevertheless, commuting bats did not reduce acoustic sensory input when visual cues were available, which highlights the importance of sonar sampling for short-range orientation in insectivorous bats. Even when there were ample visual cues (>100 lx), the bats continued to echolocate, showing that biosonar remains the primary sensory modality. Thus, common noctules, along with Egyptian fruit bats, are hardwired to maintain echolocation when orienting during commuting (8). This contrasts with other echolocating flying vertebrates, such as oilbirds and some fruit bats (9), which primarily rely on vision and can entirely dispense with echolocation during orientation. Maintaining a constant level of acoustic sensory input, irrespective of light levels, does not imply that common noctules ignore visual cues; rather, they may continually integrate information acquired by both sensory modalities simultaneously, as has been observed in captive bats navigating around obstacles (10) or through tunnels (11).

### Common Noctules Reduce Echolocation While Hunting in Light.

**Fig. 2.**
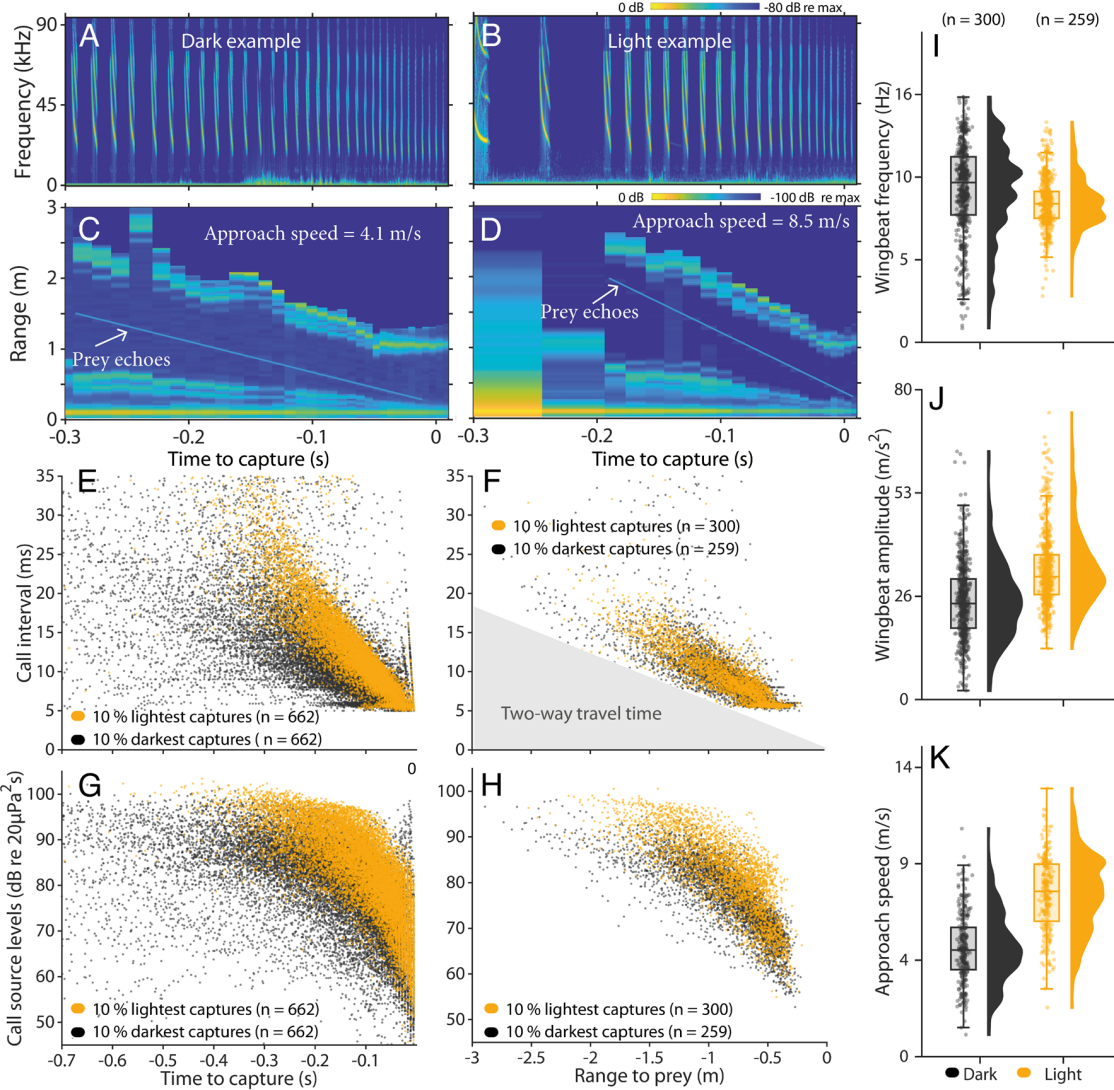
Bats reduce their use of echolocation when hunting in lit conditions. Example spectrograms (*A* and *B*) and echograms (*C* and *D*) of one hunting event in dark (*A* and *C*) and lit conditions (*B* and *D*). Call intervals (*E* and *F*) and source levels @ 10 cm (*G* and *H*) for captures during the 10% lowest and highest light levels are plotted according to time to prey interception (*E* and *H*) and range to prey (*F* and *H*) (n = 662 captures in dark and in light). The two-way travel time to and from the prey (ms) is calculated (gray) from the range to prey and the sound speed in air. Mean wingbeat frequency (*I*), amplitude (*J*), and bat–prey approach speeds (*K*) for all hunting events are plotted for the 10 % lowest and highest light levels, where we could extract the approach speeds from (n = 300 in dark and 259 in light).

We next predicted that, when tracking prey in lit conditions, bats would sample their environment at a slower rate and with higher call levels, but would not change their flight gait due to the need to use prey-specific capture techniques. To ensure that the differences in echolocation reflected changes in light levels rather than different prey types occupying light and dark environments, we compared the captures of similarly sized prey (target strengths from −41 to −20 dB at a distance of 10 cm), caught below 200 m altitude to exclude migrating insects, and away from streetlights (indicated by drastic changes in light intensity as recorded by on-board sensors). When tracking prey in light conditions, bats decreased their call intervals by 65.2 (CI: 64.7 to 65.7) ms/second of flight toward prey compared to 31.7 (CI: 31.2 to 32.1) ms/s in darkness (model 3, LMM, Fig. 2*E*). During the brightest captures, they paired such slower call rates per time with 7 dB louder call levels likely to maintain sensory redundancy while using a reduced sample rate (*t* test, SD: 7, t(39686) = −91, *P* < 0.00001, model 4, Fig. 2*G*). These findings suggest that common noctules reduce their acoustic tracking of prey when light is available, which is consistent with the idea that bats integrate visual with acoustic information when hunting prey.

### Multisensory Integration Supports Faster Tracking of Prey.

To investigate whether this bimodal sensing strategy would result in a more efficient hunting strategy, we compared wingbeat patterns and approach speeds in lit and dark conditions. When tracking prey in either condition, the wingbeat frequencies were similar (9.0 ± 1.6 in light vs 9.8 ± 2.8 Hz in darkness, Fig. 2i, model 5, GLMM, *P* = 0.76), yet bats flew with more powerful wingbeats compared to when they flew in darkness (33.4 ± 9 in light vs. 25.3 ± 9 m/s^2^ in dark, model 5, GLMM, *P* < 0.0001, Fig. 2*J*), and the bats approached their prey faster in lit than in dark conditions (7.9 ± 1.8 in light vs. 5.2 ± 1.7 m/s in dark, model 5, GLMM, *P* < 0.0001, Fig. 2*K*). Since, the bats approached their prey in light 1.5 × times faster, while relying on half the sampling rate per time to prey, they did not adjust sampling rate to time-to-capture. Instead, we found that the bats used similar reductions in call intervals relative to range [9.5 (CI: 9.15 to 9.9) ms/m in darkness to 9.6 (CI: 9.1 to 10.1) in light, model 6, Fig. 2*F*], but they produced 5 dB louder call source levels per range in lit than in dark conditions (SD: 4.8, t(7496) = −42, *P* < 0.00001, model 7, Fig. 2*H*). This shows that the bats used a consistent echolocation strategy, adjusting their call rates to match the two-way travel time to prey, regardless of ambient light conditions (Fig. 2*F*). The tagged bats thus adjusted their sonar behavior in a range-dependent manner, presumably managing faster flight speeds by supplementing their biosonar with vision demonstrating how bats can integrate flight and sensing according to light conditions. The bats may have exploited the increased visibility of their surroundings in light to fly faster, which could lead to similar changes in echolocation behavior. However, in both scenarios, whether light improves prey visibility or flight control, the bats are likely to integrate visual input into their foraging strategy. These results suggest that higher information input from multiple sensory systems contributes to better decision-making and a more efficient prey-tracking strategy involving faster and more powerful flights during insect hunting. This aligns with behavioral studies of captive fringe-lipped bats (*Trachops cirrhosis*) (12) which integrate passive and active listening to increase hunting speed in noisy conditions. Although the common ancestor of bats probably encountered challenges in aerial hunting due to its small eyes, which lead to the evolution of echolocation for hunting aerial prey (13), our study suggests that modern bats have nevertheless retained the ability to integrate vision into their hunting behavior to catch prey efficiently.

## Materials and Methods

We tagged 21 common noctules with sound, motion, and light sensors to compare their echolocation and movement behavior during commuting and prey tracking under dark and bright ambient light levels. We quantified their call intervals and levels in relation to wingbeat frequency and amplitude and used echograms to extract the approach speeds of the bats as they caught prey in air. See *SI Appendix*.

## Supplementary Material

Appendix 01 (PDF)

## Data Availability

Code and mat-files data have been deposited in Mendeley (14).
