# Supplementary material for: Wild bats hunt insects faster under lit conditions by integrating acoustic and visual information
Source: Proc Natl Acad Sci U S A. 2025 Sep 8;122(37):e2515087122. doi: 10.1073/pnas.2515087122 (PMC12452927; doi:10.1073/pnas.2515087122)
Supplement: Supplementary file 1 — Appendix 01 (PDF) [file pnas.2515087122.sapp.pdf]

Materials and Methods

Resource availability

Lead contact

Further information and requests for resources should be directed to and will be fulfilled by the lead contact, Laura Stidsholt (laura.stidsholt@bio.au.dk).

Materials availability

This study did not generate new unique reagents.

Data and Code Availability

- Data generated in this study have been deposited at Mendeley Data (DOI: https://data.mendeley.com/datasets/9rgx2z4s9b/2) and is publicly available.
- The original code has been deposited at Mendeley Data (DOI: https://data.mendeley.com/datasets/9rgx2z4s9b/2) and is publicly available.
- Any additional information required to reanalyse the data reported in this paper is available from the lead contact upon request.

Experimental model and subject details

In this study, we used already partly published acoustic, movement and light data from 21 adult common noctule bats (*Nyctalus noctula*, Vespertilionidae) tagged with sensor loggers during the summer of 2022 and 2023 in the Berlin metropolitan area (location: 52.5448, 13.5407) and in the outskirts of Rostock (location: 54.1032, 12.1341) (Stidsholt et al., 2024). All experiments were conducted under the corresponding animal welfare and conservation permits of the federal agencies: 0168/21 and IIIB293 OA-AS/FAS/705 for Berlin and 7221.3-1-057/19 and 6713WA006_19 for Mecklenburg-Vorpommern.

Method details

Experimental setup and recording equipment

Bats were equipped with sensor tags (Ocean Instruments, Auckland, New Zealand) following the procedure of (Stidsholt et al., 2024). The acoustic tag recorded audio with an ultrasonic microphone (FG-23329, Knowles Electronics, Itasca, IL, USA) and sampled the bat's behaviour by synchronised tri-axial accelerometers and magnetometers (Stidsholt et al., 2018). The audio was recorded at a sample rate of 187.5 kHz (16-bit resolution) and with a clip level of 110 dB re 20µPa. The microphone output was filtered with a one-pole, 10 kHz high-pass filter and an antialiasing filter of 80 kHz before sampling. The accelerometers sampled at 1000 Hz (16-bit resolution, 8 g clip level) with a 250 Hz anti-alias filter, while the magnetometers sampled at 50 Hz. We calibrated the accelerometer and magnetometer output data (sensu Stidsholt et al., 2018) expressing accelerometer data in m/s^2 and magnetometer data in µT. The tags also sampled ambient light with a light sensor (VEML6035, Vishay Intertechnology, Inc., Malvern, PA, USA). The light sensor sampled ambient light illuminance at 50 Hz (16-bit resolution) and with a clip level of 100 lux, a noise floor of 0.0032 lux, and a resolution of 0.0032 lux.

Tagging effects

The total unit weighed less than 10 % of the bats' body mass, which we found acceptable due to the shortness of the study, and since previous studies on the same species showed no health issues arising from this extra load (Kelling et al., 2024; Reusch et al., 2023). We cannot exclude the fact that adding 10 % of the body weight of the bats may affect the movements and flight gaits of the bats. However, since this study focused on comparing the sensory sampling when commuting and hunting in varying light conditions, any impact on the sensory sampling would affect the data consistently in dark and lit environments. We therefore find it unlikely that the tagging effects of the bats would affect the outcome of this study.

Quantification and statistical methods

Call parameters and buzz extractions in audio data

Tag data were analysed using custom software in Matlab 2023b (Mathworks). All calls were automatically extracted based on a custom-written call detector according to (Stidsholt et al., 2024). Call source levels of the off-axis calls recorded on the tags (AOLs) were estimated in energy flux density (EFD) over a 95 % energy window expressed in the unit: dB re 20µPa^{2}s. We added 14 dB to the AOLs to estimate the on-axis call source levels @ 10 cm as if they had been recorded in front of the bat (sensu (Stidsholt et al., 2018)). Since most commuting calls of common noctule bats clip the recording chain, we calculated SLs only during hunting events. We automatically detected and defined buzzes if more than four consecutive calls were below 10 ms. Buzz durations were defined as the time from the first to the last buzz call. We manually

checked all calls detected by the automatic detector within the last one second before prey captures to remove false detection and to add undetected calls. We then defined a prey capture event as the last 0.7 second before the end of each buzz. We selected a threshold of 0.7 seconds to define individual prey capture events, as longer windows (e.g., 1 second) occasionally included buzz calls from preceding capture attempts. This threshold is also supported by previous studies, which have shown that most prey capture sequences are typically completed within 0.5 seconds (Kalko, 1995; Stidsholt et al., 2021). We clustered the prey capture data into three clusters according to time before prey capture (in seconds) and the call interval (in ms) using K-means clustering (matlab function: "kmeans" and 3 clusters). We only analysed the cluster closest to the prey to include the part of the hunt with as many unclipped call source levels as possible.

Definitions of behaviours

To classify the behaviour of the bats into resting, commuting, and foraging categories, we first divided the data into 15 second segments and extracted four features per segment. From the audio data, we used the buzz rate (i.e. the number of buzzes per segment) since this parameter is useful for distinguishing between foraging and commuting flights. From the movement data, we used wingbeat frequency (Hz), the sum of the jerk (i.e. the changes in acceleration, m/s^3), and the maximum roll angle (Euler angles) of downsampled acceleration data (100 Hz). The mean wingbeat frequency per segment was defined as the peak of the spectrum (using pwelch function in matlab with a window of the entire signal and a 50 % overlap) of 2 Hz high pass filtered z-axis accelerometer data (a delay-free FIR filter using a filter length of 1024 samples). The jerk of the bat's movement is defined as the rate of change of the bat's acceleration over time, and is calculated by taking the norm to the differentiated, unfiltered three accelerometer axes (Fais et al., 2016). The roll was estimated using standard procedures (Johnson & Tyack, 2003) of 2 Hz low-pass filtered 3-axis accelerometer data (a delay-free FIR filter using a filter length of 1024 samples).
To classify the data, we used supervised machine learning through the Classification Learner App in MATLAB. For the training data, we separated the data into resting and flying based on the presence or absence of wingbeats. We then separated the flying epochs into commuting and foraging based on the presence or absence of buzzes, the maximum roll angle, and the maximum jerk of each segment. We used 5 tag recordings as a training dataset, and the remaining 16 as test data. The classifier that performed best (98.8 %) was a fine decision tree. We used this classifier to divide the data into three categories: resting, commuting, and foraging which were used to make behavioural ethograms.

Approach speed and range estimates during prey captures

We wanted to investigate the ranges to the prey at call emission and the speed at which the bats tracked and caught their prey. To do this, we extracted the time-delays between successive emitted calls and the returning prey-echoes. We can use the time-delays to estimate prey range by multiplying with the sound speed in air. From the gradual decrease in distance to the prey over time, we can reliably estimate the speed at which the bat is closing in on the prey *i.e.* the approach speed of the bat to the prey. To extract the correct echoes belonging to the prey from each call, we visualised each prey capture in echograms. Echograms are visual representations of the acoustic scene of the bats over time akin to a fish-finder plot (Johnson et al., 2004). Prey echoes were identified as a sequence of short echoes returning at gradually shorter time-intervals prior to the end of a buzz. These prey echo traces ere visible in approximately 42 % of the captures (N = 300 in darkness and 259 in light out of the 662 and 662 darkest and lightest captures). In the remaining captures, prey echoes could not clearly be identified, and we therefore discarded these captures for the approach speed analysis. We manually marked the beginning and ending of each visible prey echo trace on the echograms. The slope of the regression line between these points were used as the bat-prey approach speeds. Prey sizes (i.e. the mean target strength of each capture) were estimated from the call source levels, the returning prey echo levels and the ranges at each call emission from the prey echoes traces of the echograms following (Stidsholt et al., 2024) using the sonar equation (Urick, 1983).

Light analysis

We distinguished dark and light conditions based on two different thresholds for two different analyses. i) We first used an overall mean of the recorded light levels of ~0.6 lux across both foraging and commuting data (Fig. 1f), roughly corresponding to light under full moon. This threshold is used to compare commuting and hunting behaviours and resulted in 2800 light and 8954 dark commuting segments (n = 11754 in total), and in 4811 light and 1808 dark foraging events (n = 6619 in total) from 21 bats (Fig. g-i).

We also compared captures in darkness and in light, but to eliminate potential confounding factors from different insect families occupying different light niches, we discarded any captures with prey events below 200 meter to discard migrant species, and with target strengths between -41 and -21 dB. We then extracted the 10 % darkest (< 0.17 lux, n = 662 hunting events from 18 bats) and 10 % brightest (>37 lux, n = 662 hunting events from 15 bats) prey captures corresponding to light under a crescent moon and dim light, so to further investigate the effects of light. To analyse the differences in speed in light and darkness, we used the prey echo stream of echograms to calculate the approach speeds per capture. Prey echo streams were only possible to extract in a subset of the data (N = 300 in darkness and 259 in light).

We found no differences in the behaviour of the bats using a 10 % threshold for commuting flights, so, to include as much data as possible, we used the mean threshold for commuting data. Even though visual sensitivity and acuity vary across species and foraging strategies, most bat species respond optimally within the ranges of this study [5,6] making both visual and echolocation cues available to the tagged bats.
The light data was converted to log values by taking log10 to the lux values after adding half the resolution to the light levels to avoid log(0) issues. The light data was then averaged every 1 second during commuting flights or averaged across the one second before each prey capture.

Movement analysis
We wanted to compare the wingbeat frequency, and wingbeat amplitude across behaviours and light levels in the one second intervals of commuting flights or one second prey captures. The mean wingbeat frequency was estimated as the peak frequency of the power spectrum (nfft = 50 samples) of the 15 Hz lowpass filtered z-axis acceleration data. The wingbeat amplitude was estimated as the peak of the filtered z-axis acceleration data (using a 10 Hz lowpass delay-free FIR filter with a filter length of 100 samples). The wingbeat peaks of each segment were extracted using matlab function “findpeaks” with a minimum peak height of 2 m/s^2 and a minimum peak distance of 5 samples. We used the mean of all wingbeat peaks as the mean wingbeat amplitude per segment of either commuting flight or prey capture.

Statistics
Statistical tests were performed in MATLAB or R (version 4.3.2) using the “lme4” and “performance” packages or in MATLAB (version: R2023b) using the “fitglm” and “plotResiduals” functions. In this section, we report the fixed effects, standard errors (SE), and p-values for the model fits here, or the mean values and standard deviations.

Model 1: We first wanted to test how ambient light levels affect wingbeat interval both for commuting and foraging. We used the mean wingbeat interval per segment as response variable, the behaviour (i.e. commuting vs. foraging) and light level (i.e. dark vs. light) as categorical explanatory variables and bat ID as random effect. We fitted a linear mixed-effects model (LMM) to the data using the function “fitlme” in MATLAB. The residuals deviated slightly from the expected distribution. The model shows that the wingbeat intervals increased from 127.5 (SE: 1.9) ms during commuting in darkness to 131.2 (SE: 0.7) ms in light. When foraging, the wingbeat intervals decreased by 13 (SE: 0.8) ms (LMM, $p<0.0001$) compared to the commuting valuesl.

We then wanted to test how ambient light affected the call intervals when commuting. Since the call intervals are bimodally distributed due to the coupling to the wingbeats, we first clustered the data in two (using kmeans function in matlab). We calculated the mean and SD of each bimodal peak of the call intervals. To test whether the slight increase in call intervals in light during commuting was a passive consequence of the increased wingbeat intervals, we calculated the ratios of call intervals:wingbeat intervals in light and darkness for the first and second peak of the call intervals. For cluster 1, with mean call intervals of 125.8 (SD: 33.1) ms and 126.4 (SD: 35.9) ms, the ratio to the wingbeat interval of 131.9 and 139.2 is 1.04 and 1.1 corresponding to one call per wingbeat. For cluster 2, with mean call intervals of 276.7 (SD: 40.1) ms and 283.8 (SD: 33.1) ms, the ratios to the same wingbeat intervals are 0.48 and 0.48 corresponding to one call every second wingbeat. We therefore conclude that the increased call intervals in light during commuting is a passive consequence of slower wingbeats.

Model 2: We investigated the relationship between the mean wingbeat amplitude and the behaviour and light levels of the bats. We used the mean wingbeat amplitude as normally distributed response variable, the behaviour (i.e. commuting vs. foraging) and light level (i.e. dark vs. light) as categorical explanatory variables and bat ID as random effect. We also used an interaction term between the behaviour and light level, and

discarded 1 outlier tag recording. We fit a linear mixed-effects model (LMM) to the data using the function “lmer” in the “lme4” R package. All diagnostic plots of the performance-package fit the expected plots except the residuals that slightly deviated from the expected distribution. The model explained 62 % of the variance in the data (R-package “rsq”) of which the fixed effects explained 23 %. The fixed effects and standard errors of the model show that the wingbeat amplitude increases from 22 (SE: 1.15) m/s^2 during commuting to 26 (SE: 0.14) m/s^2 during foraging ($p<0.001$), while the fixed effect is 2 (SE: 0.12, $p<0.001$) m/s^2 for light level and 2.5 (SE: 0.2) m/s^2 for the interaction term ($p<0.001$). The model therefore shows that light has a stronger effect on the wingbeat amplitude during foraging compared to during commuting.

Model 3: We investigated the relationship between the call interval changes with **time to prey** within the last 0.7 seconds of the prey attack. We compared the 10 % darkest and brightest prey captures for the data of cluster 3 (i.e. calls with call intervals below 40 ms). We fitted an LMM to the call intervals using time to capture (N = 662 and 662 captures in darkness and in light) as explanatory variable, and bat ID as random effect. The slope of the call intervals according to time changes from -31.7 (CI: -32.1 to -31.2) ms/second in darkness to -65.2 (CI: -64.7 to -65.7) in light. Since the 95 % confidence intervals do not overlap, the bats changed their rate of call interval adjustments to time in light compared to in darkness.

Model 4: We investigated the relationship between the call source level changes with **time to prey** in the 10 % darkest and brightest prey captures for the data of cluster 3 (i.e. calls with call intervals below 40 ms, N = 662 and 662 captures in darkness and in light as in mode 3). Call source levels above 103 dB re 20µ Pa^2s are clipped, thereby affecting any fitting of the data with this effect being more pronounced for the light captures where the bats call louder. Since the bats in light call louder than in darkness, we first fitted the call source level data in darkness to log10(time) and found that the bats reduced their source levels by 34 (SE: 0.31)*log10(time). We used this linear model output on the light data to generate residual plots for the call source level adjustment according to log10(time) in light. Here we assume that in the absence of a response, the residuals should be drawn from the same distribution as in darkness (Pedersen et al., 2024). We found that the median of the residuals in light were 7 dB higher than in darkness (ttest, SD: 7, $t(39686) = -91$, $p < 0.00001$), and that the bats therefore on average called 7 dB louder in light.

Model 5: We wanted to investigate if there were any differences in the hunting behaviour between lit and dark environments (i.e. 10 % brightest and 10 % darkest prey captures, N = 300 in darkness and 259 in light, the subset data that could be extracted from echograms). We fitted a GLMM model using light treatment as a binomial response variable and wingbeat frequency, amplitude and approach speed as continuous explanatory variables, and bat ID as random effect. Wingbeat amplitudes were stronger (LMM, $p<0.0001$), while approach speeds (LMM, $p<0.0001$) significantly faster in lit conditions. Wingbeat frequencies on average decreased from 9.8 m/s in darkness to 9.0 m/s in lit conditions, however this was statistically insignificant (LMM, $p=0.76$). This contrasts with the overall data set (N = 6619 captures) where the wingbeat frequency was slower when hunting in lit conditions (model 1, LMM<0.0001).

Model 6: We investigated the relationship between the call interval changes with **range to prey** within the last 0.7 seconds of the prey attack. We compared the 10 % darkest and brightest prey captures for the subset of data where we could reliably extract echo traces off the echograms (n = 559 of 1324 captures). We fitted an LMM to the call intervals using range to capture as explanatory variable, and bat ID as random effect. The slope of the call intervals according to range changes from 9.5 (CI: 9.15 to 9.9) ms/second in darkness to 9.6 (CI: 9.1 to 10.1) in light. Since the 95 % confidence intervals overlap, the bats did not change their rate of call interval adjustments to range in light compared to in darkness.

Model 7: We tested the relationship between call source levels with **range to prey** in darkness and in light using the same data as in model 6, and the same approach as in model 4. We found that the residuals in light were 5 dB higher than in darkness (ttest, SD: 4.8, $t(7496) = -42$, $p < 0.00001$).

References

Fais, A., Johnson, M. P., Wilson, A. M., Aguilar Soto, N., & Madsen, P. T. (2016). Sperm whale predator-prey interactions involve chasing and buzzing, but no acoustic stunning. *Scientific Reports*, *6*(June), 1–13. https://doi.org/10.1038/srep28562

Johnson, M. P., Madsen, P. T., Zimmer, W. M. X., Aguilar de Soto, N., & Tyack, P. L. (2004). Beaked whales echolocate on prey. *Proceedings of the Royal Society of London. Series B: Biological Sciences*, *271*(Suppl 6), S383–S386. https://doi.org/10.1098/rsbl.2004.0208

Johnson, M. P., & Tyack, P. L. (2003). A digital acoustic recording tag for measuring the response of wild marine mammals to sound. *IEEE Journal of Oceanic Engineering*, *28*(1), 3–12. https://doi.org/10.1109/JOE.2002.808212

Kalko, E. K. V. (1995). Insect pursuit, prey capture and echolocation in pipestirelle bats (*Microchiroptera*). *Animal Behaviour*, *50*(4), 861–880. https://doi.org/10.1016/0003-3472(95)80090-5

Kelling, M., Currie, S. E., Troxell, S. A., Reusch, C., Roeleke, M., Hoffmeister, U., Teige, T., & Voigt, C. C. (2024). Effects of tag mass on the physiology and behaviour of common noctule bats. *Movement Ecology*, 1–12. https://doi.org/10.1186/s40462-024-00477-7

Pedersen, M. B., Egenhardt, M., Beedholm, K., Luo, J., Stidsholt, L., Pedersen, M. B., Egenhardt, M., Beedholm, K., Skalshøi, M. R., & Uebel, A. S. (2024). Superfast Lombard response in free-flying , echolocating bats Report Superfast Lombard response in free-flying , echolocating bats. *Current Biology*, 1–8. https://doi.org/10.1016/j.cub.2024.04.048

Reusch, C., Paul, A. A., Fritze, M., Kramer-Schadt, S., & Voigt, C. C. (2023). Wind energy production in forests conflicts with tree-roosting bats. *Current Biology*, *33*(4), 737-743.e3. https://doi.org/10.1016/j.cub.2022.12.050

Stidsholt, L., Greif, S., Goerlitz, H. R., Beedholm, K., Macaulay, J., Johnson, M., & Madsen, P. T. (2021). Hunting bats adjust their echolocation to receive weak prey echoes for clutter reduction. In *Sci. Adv* (Vol. 7). http://advances.sciencemag.org/

Stidsholt, L., Johnson, M. P., Beedholm, K., Jakobsen, L., Kugler, K., Brinkløv, S., Salles, A., Moss, C. F., & Madsen, P. T. (2018). A 2.6-g sound and movement tag for studying the acoustic scene and kinematics of echolocating bats. *Methods in Ecology and Evolution*, *October*, 1–11. https://doi.org/10.1111/2041-210X.13108

Stidsholt, L., Scholz, C., Hermanns, U., Teige, T., Post, M., Stapelfeldt, B., Reusch, C., & Voigt, C. C. (2024). Low foraging rates drive large insectivorous bats away from urban areas. *Global Change Biology*, *30*(1), 1–14. https://doi.org/10.1111/gcb.17063

Urick, R. J. (1983). *Principles of Underwater Sound* (D. Heiberg & J. Davis, Eds.; 3rd ed.). Peninsula Pub.
